# Estimating the total prevalence of PTSD among the UK police force: Formal comment on Brewin, Miller and Burchell (2022)

**DOI:** 10.1371/journal.pone.0268400

**Published:** 2022-05-20

**Authors:** Sharon A. M. Stevelink, Simon Wessely, Nicola T. Fear, Matthew Hotopf, Neil Greenberg

**Affiliations:** 1 King’s Centre for Military Health Research, Department of Psychological Medicine, King’s College London, London, United Kingdom; 2 Department of Psychological Medicine, King’s College London, London, United Kingdom; 3 Academic Department of Military Mental Health, King’s College London, London, United Kingdom; 4 South London and Maudsley NHS Foundation Trust, London, United Kingdom; University of the Witwatersrand, SOUTH AFRICA

## Introduction

We appreciate the interest and commentary from Professor Brewin, Dr Miller and Professor Burchell regarding our article published in PLOS ONE titled ‘Probable PTSD, depression and anxiety in 40,299 UK police officers and staff: Prevalence, risk factors and associations with blood pressure’ [1].

Brewin and colleagues outline a range of factors that may have influenced the discrepancy in the prevalence of PTSD reported in two papers regarding the UK police force. Our paper reported a PTSD prevalence estimate of 3.9% and 27% in those who reported exposure to a traumatic incident in the past 6 months (14.5% of the sample) [1]. Brewin and colleagues (2020) found a prevalence 20.6% (PTSD and complex PTSD (CPTSD) combined)) throughout an officer’s lifetime [2]. This estimate was based among those who reported to have been trauma exposed in their capacity as a police officer (n = 10401, 85% of total sample).

First, the consideration of recruitment processes in both studies is vitally important. We used data from the Airwave Health Monitoring Study. This study was established to monitor possibly physical health impacts of a new communication system (TETRA) on police employees. Police officers were recruited via participating police forces across Great Britain. Our view is that the recruitment process used by ‘The Job, The Life Survey’ is very likely to have attracted a biased sample in a very different manner to police staff who were interested in finding out more about their health. ‘The Job, The Life Survey’, led by Prof Brewin and colleagues, recruited opportunistically via social media, via official police channels asking for ‘willing volunteers’ and featured prominently on a website of a policy charity with a focus on the physical and psychological welfare of police staff. We suggest that recruiting a representative sample through these means is very unlikely. For example, it is well known that social media recruitment often leads to inflated reported rates of ill-health, as those who are not feeling well are more likely to respond. To illustrate this point, when we carried out a study in military veterans, asking for personnel who had and had not been involved in morally challenging events, publicised through many routes including social media, we found that the PTSD estimates in this sample were in excess of 50% [3]. This is near 10 times the estimates we found in a representative sample of military veterans [4]. Also, it is not clear how the researchers ascertained that respondents to ‘The Job, The Life Survey’ were actual police officers and staff. People may respond to study calls for many reasons unrelated to being a member of the targeted participant pool.

Second, another factor to consider is the possible influence of a framing effect [5]. Promotion of the ‘The Job, The Life Survey’ indicated that the survey would cover ‘trauma management, wellbeing and working conditions’. Whereas the exact wording of the messaging used was altered, and as the authors indicated may have diluted a possible framing effect, this was the main aim of the survey and the survey content. Only a few mental health measures were included in the Airwave study questionnaire as it mainly focused on the use of TETRA and mobile phone, work environment, lifestyle and physical health outcomes, in addition to a health screen.

Third, we question whether the use of lifetime reporting is a sound approach to establish trauma exposure as done by Brewin and colleagues in their paper (e.g. ‘in your work with the police, have you ever experienced events which were to some extent traumatic’?). If a participant answered positively, the International Trauma Questionnaire was asked, asking the participants to refer to their most disturbing event when filling this in. We do not believe that the study design of the ‘The Job, The Life Survey’, being cross-sectional in nature, can overcome this issue. This proof can only come from longitudinal studies which ask the same questions over time. Indeed, papers on military samples show fluctuations in trauma exposures despite similar questions asked in the same sample [6, 7] as well as in non-occupational samples [8, 9]. Further, it is also well known that people’s current mental state very much affects how they recall past events (e.g. traumatic or not). Whilst it is likely that recall for events in the past six months is relatively stable, the same is not true for lifetime events which are more likely to be rated as traumatic by respondents who have a current mental disorder or poorer perceived health than those who do not [6, 7]. This is especially pertinent because it appears that over 40% of police officers in the ‘The Job, The Life Survey’ reported that the disturbing event had occurred more than 5 years in the past.

On the other hand, in the Airwave study only those who reported to have experienced a traumatic event in the past 6 months were asked the Trauma Screen Questionnaire. As rightly pointed out by Brewin and colleagues, and acknowledged in the discussion of our paper, this could have led to an underestimation of the PTSD prevalence in our sample as research shows that PTSD may take time to manifest itself, in combination with its chronicity. However, there is ample evidence within DSM-5 and elsewhere that the vast majority of PTSD symptoms develops within 6 months of a trauma [10].

We note that the authors infer that CPTSD is likely to derive from police service. This is of course possible, but we suggest that it is also quite possible (if not likely) that many people who join the police will, like the military, have a moderate or more degree of childhood adversity which may cause, or at the very least predispose them to, CPTSD. It is very important, in our view, not to make attributions of CPTSD to work experiences without further study.

Brewin and colleagues went the extra length to try and replicate our statistical analyses by only including those who reported a disturbing event within the past 6 months, and this led to nearly similar prevalence estimates. Whereas we agree with Brewin and colleagues that this does give an indication were some of the discrepancies may have come from, the other contributory factors that have influenced who and how participants responded to the mental health questions asked, have not been resolved.

Taking the findings of both studies into account we conclude that despite different prevalent estimates of PTSD, continued monitoring of the health and wellbeing of police officers is imperative. Adequate care and support should be made available to those in need, and in particular to those with recent trauma exposure.
